# Addressing the Gap: Racial Disparities and Public Health Strategies in the Epidemiology of Gastrointestinal Stromal Tumors

**DOI:** 10.7759/cureus.61743

**Published:** 2024-06-05

**Authors:** Rajmohan Rammohan, Melvin Joy, Sai Greeshma Magam, Wing Hang Lau, Dilman Natt, Abhishek Tadikonda, Sai Reshma Magam, Leeza Pannikodu, Jiten Desai, Rucha Jiyani, Saher Sheikh, Sandra Gomez, Susan Bunting, Prachi Anand, Krishnaiyer Subramani, Paul Mustacchia

**Affiliations:** 1 Gastroenterology, Nassau University Medical Center, East Meadow, USA; 2 Internal Medicine, Nassau University Medical Center, East Meadow, USA; 3 Gastroenterology and Hepatology, Nassau University Medical Center, East Meadow, USA; 4 Rheumatology, Nassau University Medical Center, East Meadow, USA

**Keywords:** healthcare equity, healthcare gap, pearson correlation coefficient, pearson correlation, linear regression analysis, linear regression, race inequities, race, gist

## Abstract

Background

Gastrointestinal stromal tumors (GISTs) represent the most common mesenchymal neoplasms of the gastrointestinal tract, arising from the interstitial cells of Cajal. These tumors bridge the nervous system and muscular layers of the gastrointestinal tract, playing a crucial role in the digestive process. The incidence of GISTs demonstrates notable variations across different racial and ethnic groups, underscoring the need for in-depth analysis to understand the interplay of genetic, environmental, and socioeconomic factors behind these disparities. Linear regression analysis is a pivotal statistical tool in such epidemiological studies, offering insights into the temporal dynamics of disease incidence and the impact of public health interventions.

Methodology

This investigation employed a detailed dataset from 2009 to 2020, documenting GIST incidences across Asian, African American, Hispanic, and White populations. A meticulous preprocessing routine prepared the dataset for analysis, which involved data cleaning, normalization of racial terminologies, and aggregation by year and race. Linear regression models and Pearson correlation coefficients were applied to analyze trends and correlations in GIST incidences across the different racial groups, emphasizing an understanding of temporal patterns and racial disparities in disease incidence.

Results

The study analyzed GIST cases among four racial groups, revealing a male predominance (53.19%) and an even distribution of cases across racial categories: Whites (27.66%), Hispanics (25.53%), African Americans (24.47%), and Asians (22.34%). Hypertension was the most common comorbidity (32.98%), followed by heart failure (28.72%). The linear regression analysis for Asians showed a decreasing trend in GIST incidences with a slope of -0.576, an R-squared value of 0.717, and a non-significant p-value of 0.153. A significant increasing trend was observed for Whites, with a slope of 0.581, an R-squared value of 0.971, and a p-value of 0.002. African Americans exhibited a moderate positive slope of 0.277 with an R-squared value of 0.470 and a p-value of 0.201, indicating a non-significant increase. Hispanics showed negligible change over time with a slope of -0.095, an R-squared value of 0.009, and a p-value of 0.879, suggesting no significant trend.

Conclusions

This study examines GIST incidences across racial groups, revealing significant disparities. Whites show an increasing trend (p = 0.002), while Asians display a decreasing trend (p = 0.153), with stable rates in African Americans and Hispanics. Such disparities suggest a complex interplay of genetics, environment, and socioeconomic factors, highlighting the need for targeted research and interventions that address these differences and the systemic inequalities influencing GIST outcomes.

## Introduction

Gastrointestinal stromal tumors (GISTs) emerge as the predominant mesenchymal tumors within the gastrointestinal tract, marking a significant area of concern within oncological studies [1]. These tumors originate from the interstitial cells of Cajal, situated at a critical junction between the nervous system and the muscular layers of the gastrointestinal tract [2]. These cells play a pivotal role in coordinating the digestive process, acting as pacemakers that regulate the contractions of the digestive tract’s smooth muscles.

The incidence and outcomes of GISTs have been observed to vary significantly across different racial and ethnic groups [1]. This variation underscores the necessity of delving into the complex interplay of genetic predispositions, environmental exposures, and socioeconomic factors that may influence these disparities. Acknowledging such disparities is crucial for developing more tailored and effective interventions to bridge the gap in GIST outcomes among diverse populations.

Linear regression analysis is a critical statistical tool in epidemiological research, especially pertinent in the study of diseases such as GISTs [3]. This analysis method allows researchers to identify points in time where significant changes in disease incidence or outcome trends occur. By detecting these “points,” researchers can better understand the dynamics of disease progression over time and evaluate the effectiveness of public health interventions. This methodology is instrumental in dissecting complex trends in cancer epidemiology, providing insights into the impact of advancements in diagnosis and treatment, as well as changes in disease prevalence.

## Materials and methods

Study design and data collection

This study explored the incidence trends of GISTs across various racial groups over a specific period, employing a detailed dataset segmented by race (Asian, African American, and White), year, and incidence counts. By aggregating data meticulously for granular analysis, the research aimed to uncover temporal patterns and racial disparities in GIST incidence rates. The methodology included comprehensive statistical analysis using linear regression models and Pearson correlation coefficients to assess the relationship between time and incidence rates among the different racial demographics, facilitating a deeper understanding of GIST incidence trends and their variations across racial groups.

Data collection and preprocessing

The primary dataset for this study, compiled from medical records using International Classification of Diseases 10th revision codes from our hospital between 2009 and 2020, patient registries, and epidemiological surveys, documented GIST incidences across four racial categories, namely, Asians, African Americans, Hispanics, and Whites, over several years to analyze temporal trends. Each data entry included the observation year, the patient’s racial background, and an incidence marker for GIST occurrences. The dataset was rigorously preprocessed to prepare for analysis, involving data cleaning to eliminate inconsistencies or incomplete records, normalization of racial categories for terminology standardization, and data aggregation by year and race. This aggregation summed up incidence markers per racial group annually, providing a consolidated view for in-depth statistical analysis of GIST incidences across different racial demographics and over time.

The determination of the study’s power and sample size was meticulously conducted through a priori power analysis, employing statistical software to process inputs derived from preliminary data or studies of a similar nature. This analytical approach aimed to estimate the minimum sample size necessary to discern an effect of a predefined size, while also adhering to a preset significance level and desired statistical power. Key considerations in this process included the anticipated data variability, the clinical significance of the effect size, the standard deviation of measurements, and the alpha level to manage the probability of type I error. By setting these parameters, the software provided an estimate for the smallest number of participants required to confidently detect the targeted effect, thereby ensuring the study was primed to fulfill its research aims efficiently. This strategic phase was paramount in achieving a balance between scientific accuracy and the practicalities of conducting research, guiding the study design to be both effective and resource-conscious.

Statistical analysis

The cornerstone of our statistical analysis hinged on two pivotal methodologies, namely, linear regression analysis and Pearson correlation coefficients. These methodologies were meticulously chosen to elucidate the trends and relationships within the data, offering a dual lens through which to examine the incidence of GIST across racial demographics over time.

Linear Regression Analysis

Linear regression was employed to model the relationship between the year of diagnosis (independent variable) and the number of GIST incidences (dependent variable) for each racial group. This approach allowed for estimating trends over time, offering insights into whether incidences increased, decreased, or remained stable. The linear regression model yields several vital outputs [4]. The slope, or coefficient, quantifies the annual change in GIST incidences, signifying whether the trend is increasing (positive slope) or decreasing (negative slope). The intercept denotes the anticipated number of incidences at the beginning of the observation period, providing a foundational baseline for assessing changes. Meanwhile, the R-squared value measures the model’s fit to the data by representing the fraction of variance in the dependent variable that can be predicted from the independent variable, indicating the model’s explanatory power.

Pearson Correlation Coefficient

Pearson correlation coefficients were calculated for each racial group to complement the linear regression analysis, assessing the strength and direction of the linear relationship between time and GIST incidences. This coefficient ranges from -1 to 1, where values close to 1 or -1 indicate a strong positive or negative correlation, respectively, and values near 0 suggest no linear correlation. A key aspect of this analysis is the p-value associated with each correlation coefficient, which tests the hypothesis that no correlation exists. A low p-value (typically <0.05) rejects this null hypothesis, affirming the significance of the correlation observed [5].

The dual application of linear regression and Pearson correlation provided a robust framework for analyzing the GIST incidence data. Linear regression models illuminated the trends within each racial group, identifying statistically significant increases or decreases in incidences over time. Meanwhile, Pearson correlation coefficients offered a nuanced view of the relationship between time and incidences, reinforcing the regression analysis findings with correlation strength and statistical significance measures.

Graphical analysis

The results from both the linear regression analysis and Pearson correlation coefficients were interpreted to glean insights into the incidence trends of GIST among the studied racial groups. These quantitative findings were complemented by graphical analyses, including scatter plots with lines of best fit for each group, visually depicting the relationship between the year and incidence counts. The graphical representations were instrumental in illustrating the statistical findings, making the trends more accessible and understandable.

Data analysis

The data were processed using SPSS for Windows (IBM Corp., Armonk, NY, USA) and RStudio software (RStudio, PBC, Boston, MA, USA). Categorical data were represented by the number of patients (n), whereas continuous data were displayed as the mean value ± the standard deviation. The p-value for the study was <0.05.

## Results

The dataset encompassed the period from 2009 to 2020, providing an in-depth examination of GIST cases among four racial groups, highlighting temporal patterns and the breadth of cases observed. A slight male majority was noted within the cohort, with males representing 53.19% and females 46.81% of the cases. The racial distribution of GIST cases was notably even, with Whites at 27.66%, Hispanics at 25.53%, African Americans at 24.47%, and Asians at 22.34%, indicating a well-balanced representation across racial lines and enabling a detailed analysis of GIST incidences by racial background.

The data uncovered hypertension as the predominant condition among those diagnosed with GIST, affecting around 33%, with heart failure not far behind, impacting 29% of the patients. The prevalence of other conditions such as hyperlipidemia, chronic obstructive pulmonary disease (COPD), anemia, and coronary artery disease ranged between 10% and 15%. In contrast, diabetes, stroke, smoking, liver disease, and drug abuse were notably less common, while obesity and alcohol abuse were also less prevalent. These statistics offer critical insights into the health profiles of individuals with GIST, with hypertension emerging as the most common comorbidity, followed by heart failure. Lesser rates of hyperlipidemia, COPD, anemia, and coronary artery disease were observed. In contrast, conditions such as diabetes, stroke, smoking, liver disease, and drug abuse were minimal, reflecting specific health patterns among the GIST-diagnosed population, as shown in Table 1.

**Table 1:** Demographics of the study population.

Demographic/Comorbidity	Percentage (%)
Race	
White	27.66
Hispanics	25.53
African American	24.47
Asian	22.34
Gender	
Male	53.19
Female	46.81
Comorbidities	
Diabetes	1.06
Hyperlipidemia	10.64
Hypertension	32.98
Kidney failure	8.51
Anemia	8.51
Coronary artery disease	9.57
Chronic obstructive pulmonary disease	14.89
Obesity	5.32
Alcohol abuse	4.26
Heart failure	28.72

Utilizing a combination of linear regression models and Pearson correlation coefficients, the study aimed to elucidate the temporal patterns of GIST incidence and evaluate the statistical significance of these trends across four racial categories: Asians, African Americans, Hispanics, and Whites.

**Figure 1:**
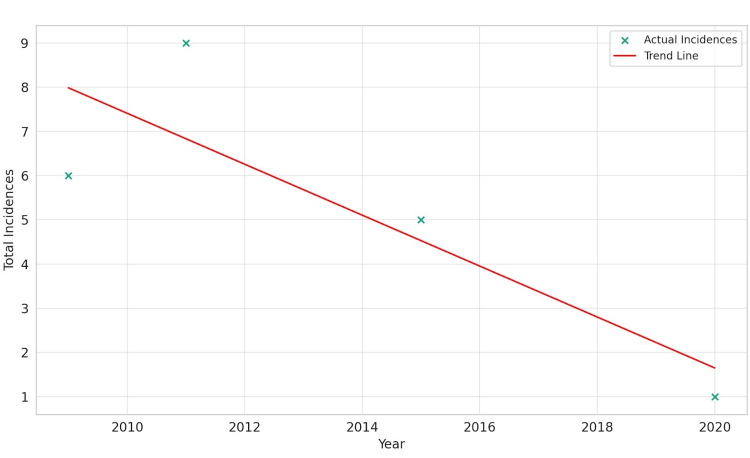
Gastrointestinal stromal tumor incidence over time for Asians.

The linear regression analysis for Asians indicated a negative slope of -0.576 with an R-squared value of 0.717. This suggests a pronounced downward trend in GIST incidence among the Asian population over the observed period, implying that GIST decreases as time progresses. The Pearson correlation coefficient for this group further reinforced this finding with a value of -0.847, indicating a strong negative correlation between time and GIST incidence. However, the p-value of 0.153 suggests that while the trend is clear, it may not be statistically significant, indicating a need for caution in interpreting these results, as shown in Figure 1.

**Figure 2:**
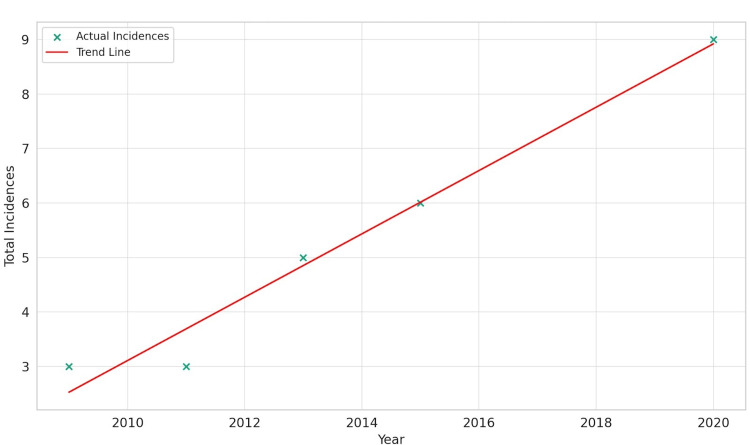
Gastrointestinal stromal tumor incidence over time for Whites.

Conversely, the data for Whites presented a starkly different picture. The linear regression model revealed a positive slope of 0.581 and an exceptionally high R-squared value of 0.971. This indicates a very strong and significant upward trend in GIST incidence among White individuals, suggesting that the rate of GIST incidence is increasing with time in this demographic. The Pearson correlation coefficient of 0.985, with a statistically significant p-value of 0.002, confirms the reliability and significance of this upward trend, highlighting a pressing public health concern that warrants further investigation and targeted interventions, as shown in Figure 2.

**Figure 3:**
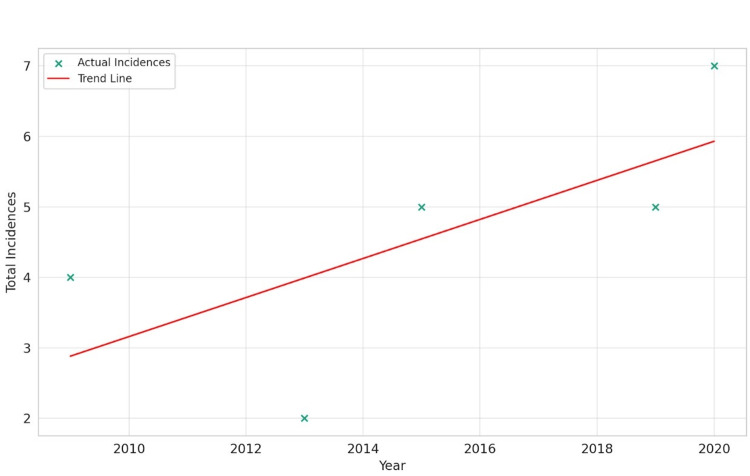
Gastrointestinal stromal tumor incidence over time for African Americans.

The analysis of African American data revealed a moderate positive slope of 0.277 in the linear regression model with an R-squared value of 0.470, suggesting a moderate increase in GIST incidence over time. The Pearson correlation coefficient of 0.686 supports a positive trend, although the p-value of 0.201 indicates that this finding is not statistically significant. This underscores the potential variability in the data and suggests that while there might be an upward trend, it is not as pronounced or statistically robust as observed in other groups, as shown in Figure 3.

**Figure 4:**
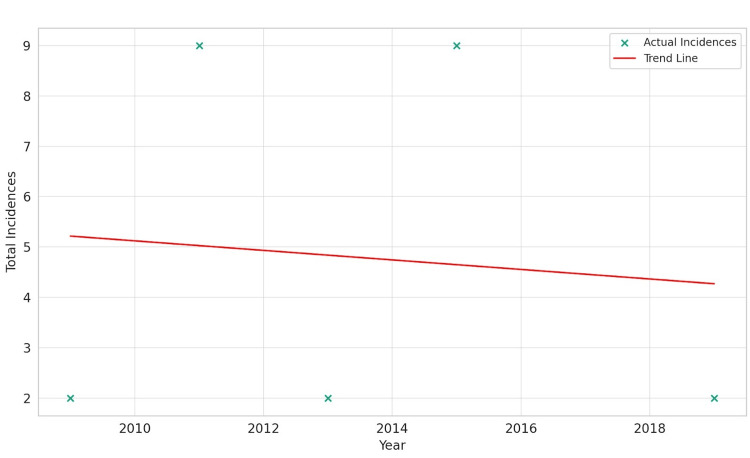
Gastrointestinal stromal tumor incidence over time for Hispanics.

Hispanics exhibited a unique pattern. The linear regression analysis showed a slight negative slope of -0.095 and an R-squared value close to zero (0.009), indicating a negligible change in GIST incidence over time. The Pearson correlation coefficient for this group was -0.095, with a p-value of 0.879, suggesting that any observed trend is not statistically significant. This finding highlights the relative stability of GIST incidence within the Hispanic population over the examined period, contrasting with the more dynamic trends observed in other racial groups, as shown in Figure 4.

The scatter plots and trend lines generated for each racial category visually encapsulate these findings, clearly representing the temporal trends in GIST incidence. The distinct trajectories observed across different racial groups underscore the complexity of GIST epidemiology and the influence of racial demographics on disease incidence rates.

The statistical significance of these trends varies, with Whites exhibiting a clear and significant increase in GIST incidence over time. These findings underscore the importance of adopting a nuanced, race-aware approach in the study of GIST epidemiology, which could inform targeted public health strategies and research priorities. Further studies are needed to explore the underlying factors driving these trends and to develop effective interventions tailored to the specific needs of each racial group.

## Discussion

Incidence and prevalence of GISTs among different racial and ethnic groups

A study examining the Surveillance, Epidemiology, and End Results (SEER) database for GISTs from 2002 to 2015 highlighted significant racial disparities in incidence and survival rates in the United States [1]. Among 7,204 GIST patients, 68.4% were White, 18.2% were African American, and 13.4% fell into an Other category, including Asian/Pacific Islander and American Indian/Alaskan Native. The overall incidence rate was 0.75 per 100,000, with African Americans experiencing the highest rate at 1.37 per 100,000 compared to Whites at 0.65 and Asians/Pacific Islanders at 1.10 per 100,000 [1]. The rate for American Indians/Alaskan Natives was the lowest at 0.28 per 100,000 [1]. The incidence for African Americans was more than double that of Whites (rate ratio [RR] = 2.12; p < 0.001), and Whites were more likely than African Americans to receive surgical treatment [6]. This study underscores the urgent need for addressing these racial disparities in GIST management and outcomes.

Studies highlighting racial disparities in GIST incidence

Research utilizing the National Cancer Institute’s SEER database has revealed racial disparities in the incidence of GISTs. Ma et al. showed that African American and Asian/Pacific Islander populations have higher GIST incidences compared to White populations [7]. These results align with earlier research spanning from 1992 to 2000, which also highlighted a higher incidence among African Americans [8], including a specific finding of increased incidence of malignant GISTs smaller than 2 cm in this group [9]. Contrarily, a study by Bangolo et al. indicated a shift over the last decade, with non-Hispanic Whites experiencing a higher incidence rate, reversing the trend observed in the previous two decades [10]. This recent development suggests the influence of other, possibly changing, factors behind these racial disparities in GIST incidence.

Factors that may contribute to disparities

Disparities in GIST incidence are multifaceted, rooted in genetic predispositions, socioeconomic status, and healthcare accessibility. The genesis of most GIST tumors involves mutations such as those in *KIT* or *PDGFRA*, with over 80% of GISTs exhibiting a *KIT* mutation. Additionally, a subset exists as wild-type GISTs, lacking detectable *KIT* or *PDGFRA* mutations [10]. Research, including findings from the SEER database, highlights the impact of socioeconomic factors on GIST incidence and outcomes. African Americans, for example, face a notably higher incidence rate of almost double that of Whites and are diagnosed at younger ages [1,11]. Age and race, particularly being older and African American, are linked with higher mortality rates [12].

Treatment disparities also play a critical role. Imatinib, a tyrosine kinase inhibitor crucial for treating and preventing GIST recurrence, underscores the need for equitable access to treatment. Its efficacy in inhibiting *KIT* and *PDGFRA* mutations enhances survival, particularly in high-risk patients, emphasizing the importance of addressing disparities in medication access due to socioeconomic barriers such as insurance coverage [1]. Moreover, marital status influences survival rates, with married individuals showing higher survival and widowed patients facing increased mortality, further illustrating the complex interplay of factors affecting GIST disparities [12].

Impact on treatment and outcomes based on race

Evidence increasingly indicates that minority ethnic groups often receive lower-quality healthcare compared to their White counterparts [13]. Specifically concerning GISTs, a 2019 study highlighted significant disparities in incidence and survival rates among different racial groups [1]. This research calls for intensified efforts to enhance GIST outcomes, emphasizing the importance of race in these disparities [1]. The interaction between patients and physicians regarding treatment options is crucial, with the race of the patient playing a significant role in these discussions [13]. Although both African American and White physicians recognize the primacy of medical information in treatment decisions, a study from 2011 found that African American physicians are more likely to consider the patient’s race as an influential factor in determining treatment choices compared to their White colleagues [14]. This difference underlines the critical need for effective communication between physicians and patients and acknowledges the impact of racial considerations on treatment decisions [15]. Such disparities and the emphasis on race in healthcare decisions point to the broader issue of ensuring equitable care and treatment outcomes for all patients, regardless of their racial or ethnic background [15].

Identification of potential interventions to reduce racial disparities in GIST care

Before 2000, disparities in the treatment of GISTs were markedly pronounced, with African Americans less likely to undergo surgery and facing higher mortality rates [6]. Since the turn of the millennium, however, this trend has notably reversed [6]. Increased access to surgical resection, along with a decrease in perioperative mortality and an enhancement in long-term survival rates for African American patients, has significantly narrowed the racial gap in GIST treatment outcomes [6]. This shift suggests a movement toward more equitable healthcare delivery in this domain [6].

Addressing the systemic causes of healthcare inequalities requires a shift in focus from individual-level risk factors to the broader, systemic factors that underpin such disparities [16,17]. Fundamental Cause Theory offers a lens through which to view health inequalities, tracing their roots back to social inequalities, including the entrenched racial hierarchies present in the United States [16,17]. This theory asserts that without tackling these foundational social inequalities, advances in medical treatment and technology may inadvertently exacerbate existing health disparities [16,17]. This is because individuals with more resources than those who are disproportionately White enjoy better access to high-quality care and cutting-edge medical interventions, owing to advantages such as living closer to top-tier medical facilities, possessing private health insurance, attaining higher education levels, and having connections within the medical community [16,17].

To truly eradicate health disparities, it is crucial to confront and address the systemic injustices embedded within the U.S. healthcare system and the broader societal fabric [18]. Efforts to implement system-level interventions have shown promise, highlighting the importance of community-level investments in key areas such as equitable education, housing, employment, and preventive and primary healthcare [18]. These initiatives are most effective when they are driven by community partnerships that not only meet social needs but also garner political support for such investments [18].

Moreover, adopting clinical training and practice approaches that emphasize structural competency can reorient health professionals and systems toward collaborating with community organizations and sectors beyond healthcare, such as housing, education, legal aid, and urban planning [19]. This collaborative approach is essential for dismantling the structural barriers to health equity [19]. It is through such comprehensive and coordinated efforts that the goal of eliminating racial and ethnic health disparities can be achieved, paving the way for a more just and equitable healthcare landscape [19].

This investigation highlights a range of limitations that could influence the breadth and applicability of its conclusions. The research delves into the incidence of GISTs across different racial groups between 2009 and 2020, utilizing methods such as linear regression and Pearson correlation for analysis. It identifies notable variances in GIST incidences across racial lines, increases in White populations, decreases among Asians, and mild shifts in African Americans and Hispanics. The study highlights the significant influence of racial demographics on GIST epidemiology, emphasizing the urgency for public health strategies specifically designed to address these disparities. It proposes that these disparities arise from a multifaceted interaction of genetic, environmental, and socioeconomic factors. The results advocate for additional research to thoroughly understand these interactions and formulate precise interventions. Furthermore, the study signals the critical need to tackle systemic healthcare disparities to enhance GIST outcomes universally, stressing on equal access to treatment and the role of socioeconomic elements in health disparities.

## Conclusions

This study offers a comprehensive analysis of GIST incidence across different racial groups from 2009 to 2020, uncovering significant disparities. It finds that GIST incidence rates vary considerably among racial groups, with Whites experiencing an increase, Asians seeing a decline, and African Americans and Hispanics showing moderate changes. This variation underscores the significant impact of racial demographics on GIST epidemiology and highlights the urgent need for public health interventions that address these disparities. The research suggests that these disparities are the result of a complex interplay of genetic, environmental, and socioeconomic factors, indicating the necessity for further investigation into these dynamics to develop targeted and effective interventions. Moreover, the study points to the critical need to address systemic healthcare inequalities to improve outcomes for all racial and ethnic groups. It emphasizes the importance of equitable treatment access and highlights the influence of socioeconomic factors on health disparities. This call to action for more race-aware research and public health strategies is crucial for bridging the gap in GIST outcomes among diverse populations, underscoring the need for systemic change to ensure equitable healthcare for all.
